# Controlling Fano resonances in multilayer dielectric gratings towards optical bistable devices

**DOI:** 10.1038/s41598-018-34787-9

**Published:** 2018-11-06

**Authors:** Thu Trang Hoang, Quang Minh Ngo, Dinh Lam Vu, Hieu P. T. Nguyen

**Affiliations:** 1 Institute of Materials Science, Vietnam Academy of Science and Technology, 18 Hoang Quoc Viet, Cau Giay, Hanoi Vietnam; 2 Graduate University of Science and Technology, Vietnam Academy of Science and Technology, 18 Hoang Quoc Viet, Cau Giay, Hanoi Vietnam; 3 Department of Electrical and Computer Engineering, New Jersey Institute of Technology, Newark, New Jersey 07102 USA

## Abstract

The spectral properties of Fano resonance generated in multilayer dielectric gratings (MDGs) are reported and numerically investigated in this paper. We examine the MDG consisting of numerous identically alternative chalcogenide glass (As_2_S_3_) and silica (SiO_2_) multilayers with several grating widths inscribed through the structure, emphasizing quality (*Q*) and asymmetric (*q*) factors. Manipulation of Fano lineshape and its linear characteristics can be achieved by tailoring the layers’ amount and grating widths so that the proposed structure can be applicable for several optical applications. Moreover, we demonstrate the switching/bistability behaviors of the MDG at Fano resonance which provide a significant switching intensity reduction compared to the established Lorentzian resonant structures.

## Introduction

Ever since the appearance of the celebrated Fano resonance more than fifty years ago^1^, it has been well-known to be the product of the interference between the discrete state and continuum background in classical or quantum systems. To date, Fano resonance can be achieved not only in classical and quantum systems but also in the photonic structures^2^, such as quantum dots^3–5^, two dimensional (2D) planar photonic crystals^6–10^, guided-mode resonances in 2D photonic crystals, single- and multi-layer grating structures^11–19^, plasmonic nanostructures^20–25^, and metamaterials^25–29^. One of the major features of the Fano resonance is an asymmetric lineshape in the spectral response which is defined by the quality (*Q*) and asymmetric (*q*) factors. The sharp asymmetric lineshape of a Fano resonance indicates an extremely high change in response that is in a narrower range than the linewidth of the resonance itself. Such critical factor is beneficial in the design of efficient optical devices. Moreover, the asymmetric sharp lineshape of a Fano resonance provides several promising photonic device applications including filters^3–5,12–14^, modulators^6,8^, sensors^20,22^, broadband reflectors^14^, lasers^6,14,18^, and switching/bistability^7,11,15–17^.

In the context of guided-mode resonances in single-layer grating structures and photonic crystal slabs^11,13–16^, the asymmetry of Fano resonance originates from a close coexistence of resonant transmission/reflection and can be reduced to the interaction of a guided-mode in the slab waveguide (discrete state) with an external radiation (continuum background) of incident light. These structures have been known as promising designs for many optical applications due to their simple, easy fan-in/out, and their low-cost fabrication processes. Whereas in the multilayer grating structures^12,16–19^, the phase resonances are added to the structure that produce circulated modes, trapped and stopped waves, and interference between waves reflected back and forth at the guided-mode resonances. Therefore, the corresponding linewidths become significantly reduced, resulting in increased *Q*-factors. Although, metal-dielectric multilayer structures have been proposed for photo-tuning and optical filters^18,19^; there are extremely inherent losses in the metals’ visible and near infrared spectral regions. In recent works^11,16,17^, guided-mode resonances in nonlinear slab waveguide gratings and coupled nonlinear slab gratings have been studied to obtain different Fano resonances, which can be applied for efficient optical switching/bistability due to their sharps and asymmetric resonant profiles. The numerical results have shown that the performance efficiency of optical switching/bistability devices, such as incident intensity for switching and contrast (between high and low states), not only depends on the *Q-*factor but is also strongly dependent on the *q-*factor of the Fano resonant lineshape. Even though the Fano resonance has offered the potential for low input intensity and high contrast for optical switching/bistability compared with Lorentzian lineshapes at the same *Q*-factor^16^; there is not any completed investigation of the dependency of switching intensity reduction on *Q*-factor. The *q-*factor describing the interference between the resonant and non-resonant pathways can be either positive or negative values; thus changing its amplitude and sign that affect the degree of asymmetric lineshape and reversal of Fano profiles. In addition, a detailed analysis of *q*-factor can help determine the switching/bistability configurations for better performance such as low switching intensity and high contrast. To study the Fano resonant generation in depth, the spectral properties of Fano resonance are numerically investigated in this work. The multilayer dielectric gratings (MDGs) consist of various identically alternative chalcogenide glass (As_2_S_3_) and silica (SiO_2_) multilayers with several grating widths. These gratings are inscribed through structures associated with the combined effects of a guided-mode resonance of MDGs and its photonic band gap with an emphasis on *Q*- and *q*-factors. As the numbers of layers and grating widths are changed, they support the controlling of Fano resonance with well-shaped and linear characteristics. Thus, this simple design allows for easy and robust control tuning the *Q*- and *q*-factors, which can have widespread and practical applications in various electronic, photonic, and integration systems. For all-optical switching/bistability applications, this structure enables low input intensity switching/bistability behaviors. Additionally, we quantitatively show that Fano resonance-based structures provide more significant switching intensity reduction than the corresponding Lorentzian resonance structure. Furthermore, because of the transparency in the telecom wavelengths, As_2_S_3_ which is an optical material with high third-order nonlinearity coefficient and ease of synthesis in thin films, can be combined with SiO_2_ to form hybrid multilayer structures. Given the low thermal expansion coefficient of SiO_2_ and the thinness of As_2_S_3_ layer in this design, the contributions from thermal expansion of these materials are found to be negligible when high optical intensity is applied^16^.

## Computational Setup

The linear and nonlinear characteristics of the proposed structures were carried out using the finite-difference time-domain (FDTD) method combined with perfectly matched layers (PML), as implemented in the MEEP software package with subpixel smoothing for increased accuracy^30–32^.

In general, the Fano resonant lineshape in the photonic system^2,17^ is given:

1 $$R(\varepsilon )=F\,\frac{{(\varepsilon +q)}^{2}}{1+{\varepsilon }^{2}}$$

where $\varepsilon =2\,\frac{\omega -{\omega }_{o}}{{\Gamma }}$; *q* and *F* are the asymmetric and constant factors; *ω*_o_ and *Γ* are resonant frequency and linewidth at half-maximum, respectively. The *Q*-factor is defined by the ratio between *ω*_o_ and *Γ*. Equation (1) has seen widespread use in Fano resonance photonics. It suggests that there is a sharp transition from the total transmission to reflection supplied by this resonance.

**Figure 1:**
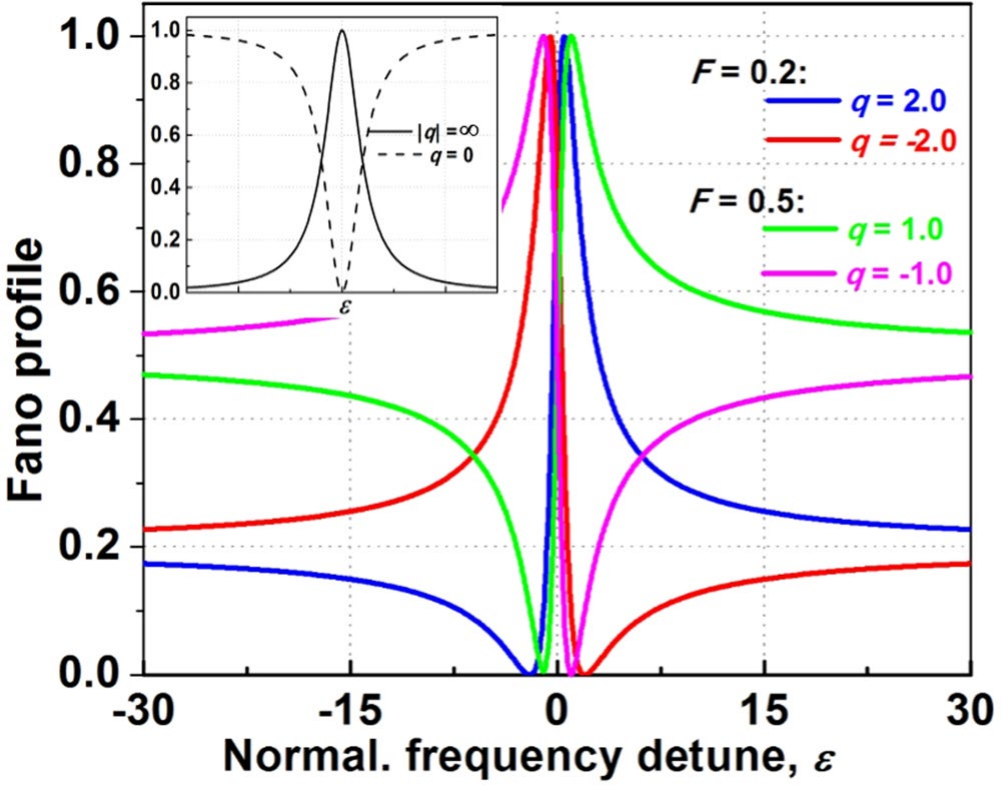
Fano profiles with the formula F*(*ε* + *q*)^2^/(*ε*^2^ + 1) for various values of the asymmetry factor *q*. The *F*-factor is chosen for maximum response of 1. The special cases are shown in the inset (Lorentzian lineshapes).

Figure 1 shows the Fano profiles according to Eq. (1) for several *q-*factors. The *F*-factor is chosen for maximum response of unity. The lineshapes for special *q*-factors are shown in the inset. As |*q*|→∞, the transition to the continuum is too weak, therefore, the lineshape is entirely determined by the transition through the discrete state only with the standard Lorentzian. As *q* = 0, it describes a symmetric dip, which are called the inverted Lorentzian lineshapes. Illustrated in Fig. 1, the degree and asymmetry of Fano lineshape depend on the *q*-factor. For |*q*| = 1, the resonant frequency is located exactly at half the distance between the peak (maximum response) and the dip (the minimum response). In addition, the reversed Fano lineshape is also plotted and the corresponding *q*-factor changes sign. As a consequence, the amplitude and sign of *q*-factor hold significant implications for various photonic devices and structures, such as high *Q*-factor filters, reflectors, lasers, detectors, sensors, as well as switching/bistability.

**Figure 2:**
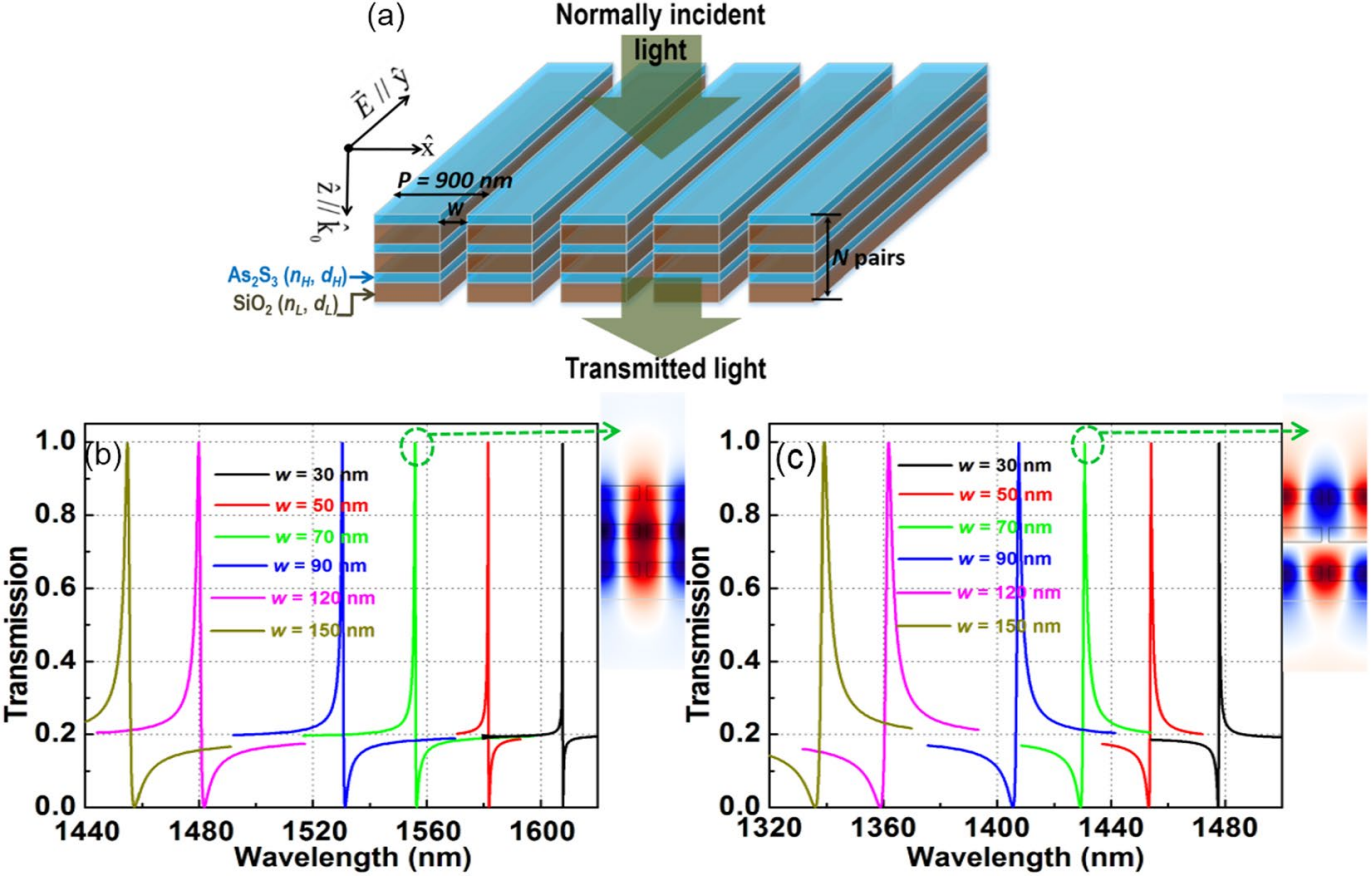
(**a**) Multilayer nonlinear dielectric grating structure with normally incident light. The structure consists of *N*-pair of bilayer As_2_S_3_/SiO_2_ gratings; the thickness of As_2_S_3_ and SiO_2_ are set at *n*_H_*d_H_ = *n*_L_*d_L_ = *λ*_center_/4, where the center wavelength *λ*_center_ is 1550 nm; (*n*_H_, *d*_H_) and (*n*_L_, *d*_L_) are the refractive index and thickness of As_2_S_3_ and SiO_2_ layers, respectively. The periodicity and width of gratings are *P* (*P* = 900 nm) and *w*, respectively; where (**b**) and (**c**) represent the enlarged regions of long and short resonances, respectively for *N* = 3. The simulations alongside (**b**) and (**c**) show field distribution at resonant peaks. TE polarization in which the electric-field is along to the strip of gratings, is used.

We present the following numerical results to demonstrate the validity and general applicability of Fano resonance in the MDG structure depicted in Fig. 2(a). It is composed of identically alternate layers of As_2_S_3_ (*n*_*o*_ = 2.38)^33^ and SiO_2_ with thickness of *t* = N*(*d*_H_ + *d*_L_) for the structure, where *N* are the repetitive identical bilayers of As_2_S_3_ and SiO_2_, and *d*_H_ and *d*_L_ are the thickness of As_2_S_3_ and SiO_2_ layers, respectively. If *N* is an odd number of layers, there is an extra As_2_S_3_ or SiO_2_ layer so the standard arrangement is with the As_2_S_3_ or SiO_2_ layers being the first and last layer. The periodicity and width of the grating structure are *P* and *w*, respectively. A transverse electric (TE) polarized normally incident plane wave means the electric field is parallel to the strip or along the *x* direction. Perfectly matched layers are set for the top and bottom sides (*z* direction) while in the *y* direction periodic boundary conditions are applied^30^. In our design, the optical thicknesses of As_2_S_3_ and SiO_2_ layers are chosen to satisfy the quarter-wavelength condition, that means *n*_H_*d_H_ = *n*_L_*d_L_ = *λ*_cent_/4, where *n*_H_ and *n*_L_ are the refractive indices of As_2_S_3_ and SiO_2_, respectively. In calculations, the center wavelength *λ*_center_ = 1550 nm, *d*_H_ = 162.8 nm, and *d*_L_ = 267.2 nm are used.

## Results and Discussion

First, we set *N* = 3 pairs of As_2_S_3_/SiO_2_ layers. The photonic band gap is obtained by using the plane-wave expansion method^34^. The calculated results show that the MDG structure has a TE band gap with the wave vector component along the z direction at 0.376 < *P*/*λ* < 0.786. Indeed, with the periodicity of gratings *P* = 900 nm, there exists the photonic TE band gap from 1145 nm to 2394 nm, in which the center wavelength 1550 nm is located in this band gap.

The transmission spectra of TE-polarized light at normal incidence for several grating widths *w* from 30 nm to 150 nm, when *N* = 3 pairs of As_2_S_3_/SiO_2_ layers are calculated as shown in Fig. 2(b,c). There exist two Fano resonances within the interested wavelength regimes, which are associated with the guided-mode resonances in the MDGs, enhanced by the photonic band gap effect along the *z* direction, due to 3 pairs of As_2_S_3_/SiO_2_ layers. The long and short resonant spectra from 1460 nm to 1610 nm and from 1340 nm to 1480 nm are shown in Fig. 2(b,c), which correspond to the TE_0_-like and TE_1_-like modes, respectively. The other TE-like transmission spectra and field profiles, such as TE_2_-like, TE_3_-like, … modes, depending on the number of layers, *N*, whose characteristics are not shown here (see Supporting Information, Fig. S1 and Table S1). The TE_2_ resonant lineshape is similar to the TE_0_ mode (same sign of *q*-factor). In addition, *Q*-factor of TE_2_ mode is smaller than that of TE_0_ mode. Similarly, with the TE_3_-like mode, it has similar lineshape and *Q*-factor smaller than TE_1_-like mode. Therefore, we ignored TE_2_- and TE_3_-like modes in switching/bistability discussion. The analytical theory of the generated high Fano resonant modes has been reported previously^35^ and is not discussed in detail here. As it is shown, the increase of grating width *w* makes the resonance shifts to the short wavelength and the *Q*-factor decreases. The spectral resonances show that the side band degrees of Fano lineshapes do not change; it even shows that the linewidths and peaks of resonances change when the grating widths change. In addition, the lineshapes of long and short resonances are reversible, which means that their *q*-factors show the opposite signs. The field profiles at long and short resonant peaks are shown in the insets, they spread along the slabs and exhibit coupling between the guided-modes and external radiations. The linear characteristics of the Fano resonances in case *N* = 3 of the long and short resonances for several grating widths *w* are summarized in Table 1.

**Table 1:** Linear characteristics of 3-pairs of As_2_S_3_/SiO_2_ grating layers for several grating widths *w*.

Grating width, *w* (nm)	30	50	70	90	120	150
**Long resonance**						
*F*-factor	0.196	0.193	0.191	0.190	0.189	0.188
Asym. factor (*q*-factor)	2.04	2.05	2.06	2.07	2.08	2.09
Resonant peak, *λ*_o_ (nm)	1607.7	1581.8	1556.0	1530.5	1480.0	1455.3
Quality factor (*Q*-factor)	17,647	6,430	3,926	2,039	1,009	772
**Short resonance**						
*F*-factor	0.185	0.187	0.189	0.191	0.192	0.193
Asym. factor (*q*-factor)	−2.09	−2.08	−2.07	−2.06	−2.05	−2.04
Resonant peak, *λ*_o_ (nm)	1477.8	1453.9	1430.4	1407.0	1361.5	1338.5
Quality factor (*Q*-factor)	4,983	2,046	1,222	860	609	544

The resonant peak *λ*_o_, constant factor *F*, quality factor *Q*, and asymmetric factor *q* are extracted by fitting Eq. (1) to the numerical spectrum. It is seen that Fano formulas mostly match the FDTD simulation results around the resonances. The interesting feature here is the similar *q*-factors as a given number of layers. For the grating widths *w* of 30 nm and 150 nm and long resonance, the Fano resonant parameters are (*λ*_o_ = 1607.7 nm, *Q* = 17,649, and *q* = 2.04) and (*λ*_o_ = 1455.3 nm, *Q* = 772, and *q* = 2.09), respectively. Whereas, for the short resonance, these are (*λ*_o_ = 1477.8 nm, *Q* = 4,983, and *q* = −2.08) and (*λ*_o = _1338.5 nm, *Q* = 544, and *q* = −2.04) for the grating widths *w* = 30 nm and 150 nm, respectively.

**Figure 3:**
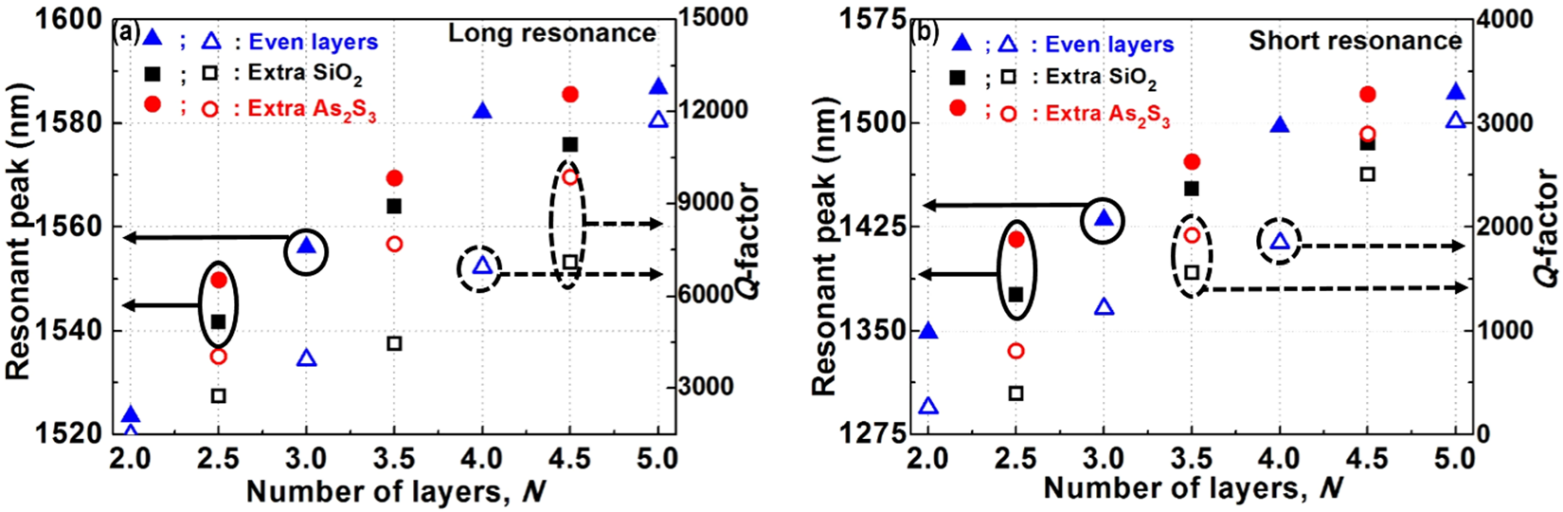
Resonant peaks and *Q*-factors of the structure as depicted in Fig. 2(a) for several number of layers *N*. (**a**) and (**b**) are with long and short resonances, respectively. The grating width *w* = 70 nm is used for these calculations. The solid (square, triangle, and circle) and hollow (square, triangle, and circle) dots are the resonant peaks and *Q*-factors, respectively.

We investigated and found that the Fano lineshapes were reproducible and readily controlled via the number of layers *N* and the grating width *w*, demonstrating the robustness of the suggested structure. With the given grating width *w* of 70 nm and for the high number of layers, the T_0_-like (long resonance) and T_1_-like (short resonance) mode linewidths are generally narrower as shown in Fig. S2; therefore, the longer optical path leads to higher *Q*-factors. In addition, increasing the effective index of the slab caused redshifts in the resonance. The resonant peaks and *Q*-factors of the long and short resonances for several number of layers *N* were evaluated using Fano lineshapes and plotted in Fig. 3. When the number of layers *N* increase, redshifts in resonance, higher *Q*-factor, and lower sidebands are obtained. For instance, with the number of layers *N* of 3 and 5, and grating width *w* of 70 nm, the resonant peak and the corresponding *Q*-factor are (*λ*_o_ = 1556.0 nm, *Q* = 3,926) and (*λ*_o_ = 1586.7 nm, *Q* = 10,684) for long resonance, respectively. Whereas, for the short resonance, these are (*λ*_o_ = 1430.4 nm, *Q* = 1,222) and (*λ*_o_ = 1521.5 nm, *Q* = 2,711) for the number of layers *N* of 3 and 5 and grating width *w* of 70 nm, respectively. These mode profiles and the resonant parameters of other modes are also shown in Table S2.

**Figure 4:**
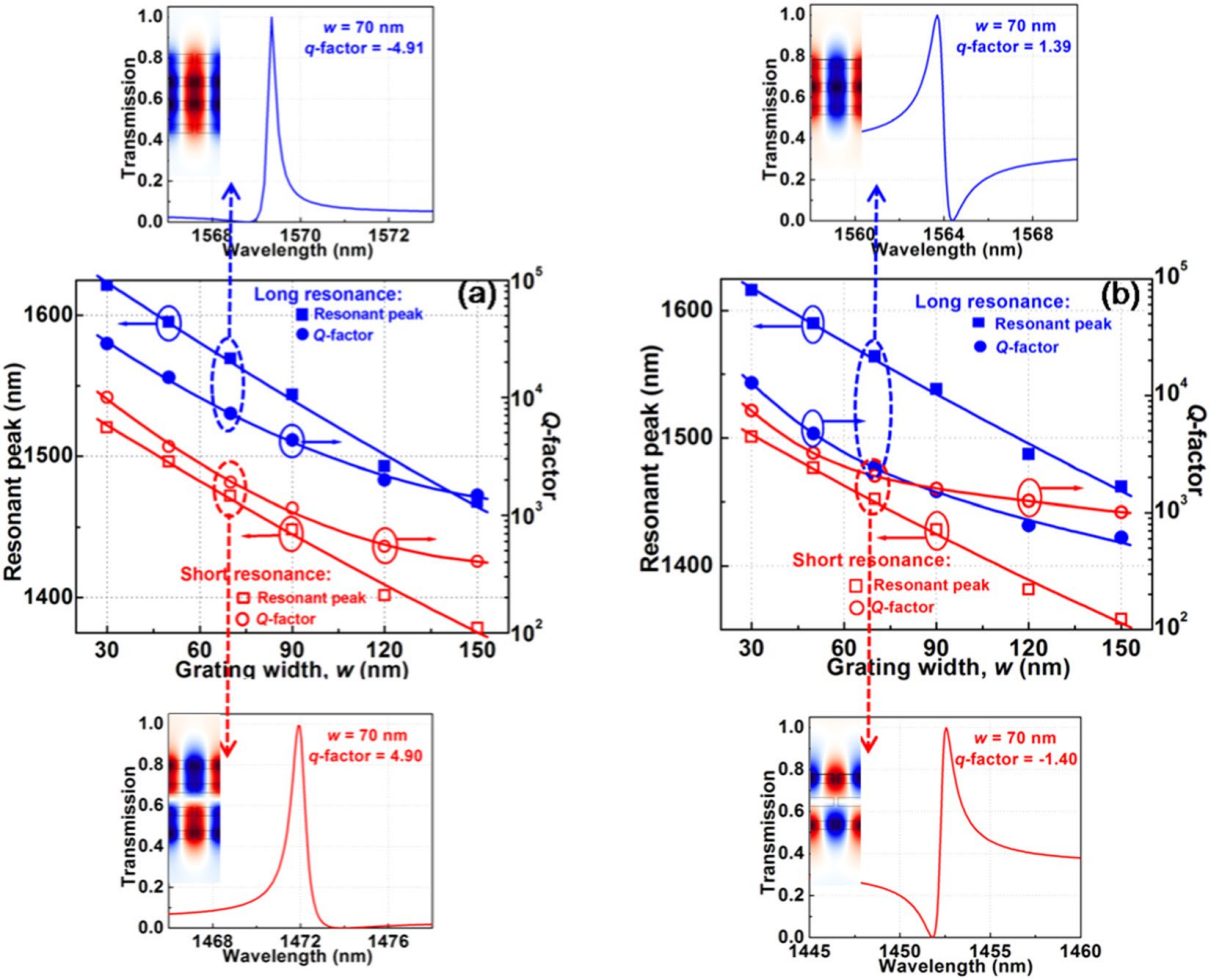
Transmission spectra of the structure as depicted in Fig. 2(a) with *N* = 3.5; (**a**) and (**b**) show the dependence of resonant peaks and *Q*-factors on the grating widths *w* of an extra As_2_S_3_ and SiO_2_ layers, respectively. The Fano resonant lineshapes for the grating width *w* = 70 nm are shown in the insets. The square and circle dots are the data points. The lines are the fitting curves.

For the results that we have shown above, the number of layers *N* was an integer. However, in the following calculation, we have studied the Fano lineshapes with a different type of *N*. With *N* = 3.5 pairs, which means the structure consists of 3 pairs of As_2_S_3_/SiO_2_ and an extra layer of As_2_S_3_ or SiO_2_ layer. The dependence of resonant peaks and *Q*-factors on the grating widths *w* for the structure are shown in Fig. 4(a,b), respectively. As seen in these figures, similar side band degrees or *q*-factors have been observed. In addition, the resonant peaks of the long and short resonances are within the range of ~1300 nm to ~1650 nm and *Q*-factors are within the range of 100 to 20,000 depending on the grating width *w*. In the insets of Fig. 4(a,b), the Fano lineshapes at resonant peaks associated with the types of structures for both long (noted by blue curves) and short resonances (noted by red curves). The insets of Fig. 4(a,b) show the field distributions at resonant peaks. They have the same behaviors as those shown in Fig. 2(b,c). The resonant peaks shift to the short wavelengths and *Q*-factors decrease as the grating width *w* increases. These are in the same tendencies as the structures of *N* = 3 pairs. With the grating width *w* of 70 nm, the resonant peaks and the corresponding *Q*-factors for various odd numbers of *N* were shown in Fig. 3.

**Figure 5:**
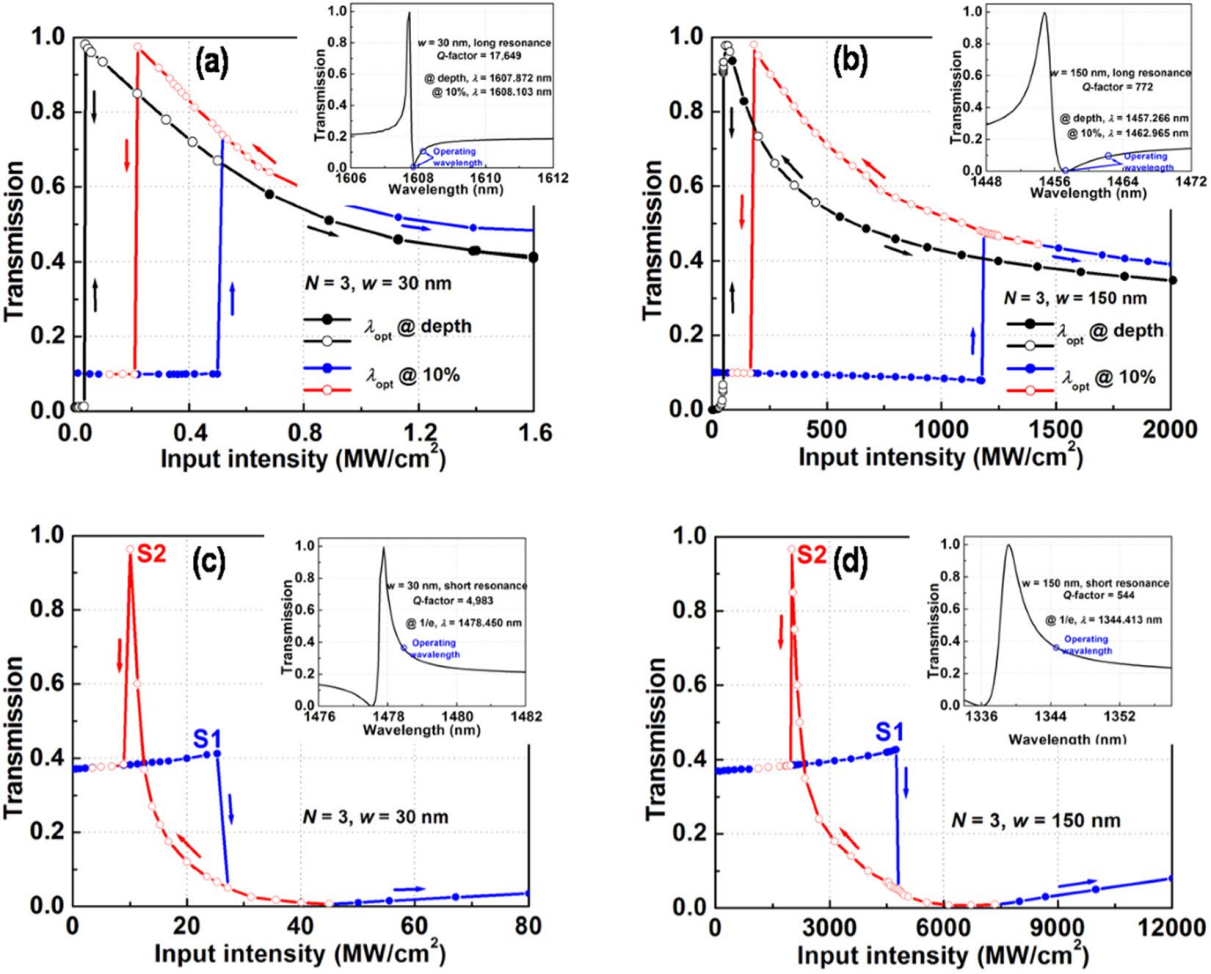
Optical switching/bistability behaviors in the nonlinear 3-pair-grating layers for grating widths *w* of 30 nm (**a** and **c**) and 150 nm (**b** and **d**) with operating wavelengths in the long (**a** and **b**) and short (**c** and **d**) resonances.

In optical switching/bistability applications^36,37^, due to the optical Kerr nonlinear effect, the refractive index of As_2_S_3_ can be modeled as *n*(*I*) = *n*_0_ + *n*_2_*I, where *n*_0_, *n*_2_, and *I* are the linear refractive index, Kerr coefficient, and intensity of incident light, respectively. In the nonlinear calculations, the third-order nonlinearity of As_2_S_3_ is *n*_2_ = 3.12 × 10^−18^ m^2^/W^38^. The refractive index increases following the intensity of the incident light, the resonant wavelength shifts to longer wavelength, so that the operating wavelength should be larger than the resonant wavelength. We investigate here the switching/bistability characteristics based on Fano resonances of the structure depicted in Fig. 2(a) with *N* = 3 pairs for both long and short resonances. The flux intensity was calculated by taking the time-averaged Poynting vector, which is an integral of $\langle {{\rm{S}}}_{{\rm{m}}}\rangle =\mathrm{Re}(\frac{1}{2}{{\rm{E}}}_{{\rm{m}}}\times {{\rm{H}}}_{{\rm{m}}}^{\ast }\,)$, where ${{\rm{S}}}_{{\rm{m}}},{{\rm{E}}}_{{\rm{m}}},{\rm{and}}\,{{\rm{H}}}_{{\rm{m}}}$ are the Poynting vector, electric field, and magnetic field phasors, respectively with the direction perpendicular to the output plane, supported by MEEP program^32^. Figure 5 shows the dependence of transmission (ratio between the transmitted and incident intensities) on the incident intensity of the optical switching/bistability for the long (Fig. 5(a,b)) and short (Fig. 5(c,d)) resonances. For the long resonance, the operating wavelengths are chosen at resonant dip and 10% of transmission as shown in the insets of Fig. 5(a,b).

The switching/bistability behaviors with one switching point are obtained. The lower branch (blue curve) is observed by varying the incident intensity of the CW source starting from low intensity. Following the lower branch, as incident intensity increases, the transmission also increases; but at the critical incident intensity the transmission increases abruptly, which is referred to the switching intensity. The higher branch (red curve) is observed by considering the modulated incident signal at high intensity (above the switching intensity) as the seed, and then the incident intensity decreasing slowly. When the incident intensity decreases over the switching intensity, the transmission sustains in the higher branch due to the feedback of the Kerr nonlinear elements^36,37^. It jumps down abruptly to the low-transmission state when the transmission reaches a unity. As shown in Fig. 5(a,b), with the operating wavelength at 10% of transmission, blue (arrows pointing up/right) and red curves (arrows pointing left/down), it shows that the bistability behaviors and the switching intensities are 0.50 MW∕cm^2^ and 1178.56 MW/cm^2^ for grating widths *w* = 30 nm and 150 nm, respectively. Whereas with the operating wavelengths at the resonant dips, the bistability behaviors have not occurred (black curves, on left) even the switching points at 0.04 MW/cm^2^ and 50.35 MW/cm^2^ of input intensities and high contrasts are observed for grating widths *w* = 30 nm and 150 nm, respectively. When the operating wavelength moves away from the resonant dip, the switching intensity is higher and the bistability region is broader. This is attributed to the wavelength detuning, which implies a broader detuning bandwidth and, thus, a higher resonance shift amount is required to change the state^16^. For the short resonance, the operating wavelength is chosen at 1/e transmission as shown in the insets of Fig. 5(c,d). In all cases, optical bistable switching behavior is clearly formed. The higher branch (blue curve, with S1) and lower branch (red curve, with S2) are observed by increasing and decreasing the incident intensities starting from low and high intensities, respectively. In fact, there exists two switching points of the optical bistable switching behaviors at the increasing (S1) and decreasing (S2) input intensities. The presence of S1 and S2 and the mechanism of this bistability behavior in these cases were discussed in^16^. In each bistable curve, the switching intensity can be estimated as the input intensity for which the transmission decreases abruptly in the blue and red curves. For example, in Fig. 5(c,d), the estimated switching intensities are 25.07 MW∕cm^2^ and 4757.90 MW∕cm^2^ (at point S1) and 10.15 MW∕cm^2^ and 2032.77 MW∕cm^2^ (at point S2) for the grating widths *w* = 30 and 150 nm, respectively. The switching/bistability characteristics for the operating wavelength at 50% transmission of the short resonance are not shown here, because they do not show the bistability behaviors. Other grating widths (*w* = 50 nm, 70 nm, 90 nm, and 120 nm), whose switching/bistability behaviors for the operating wavelengths at resonant dip and 10% transmission for long resonance and 1/e for short resonance (not shown here) exhibit the same tendency.

**Figure 6:**
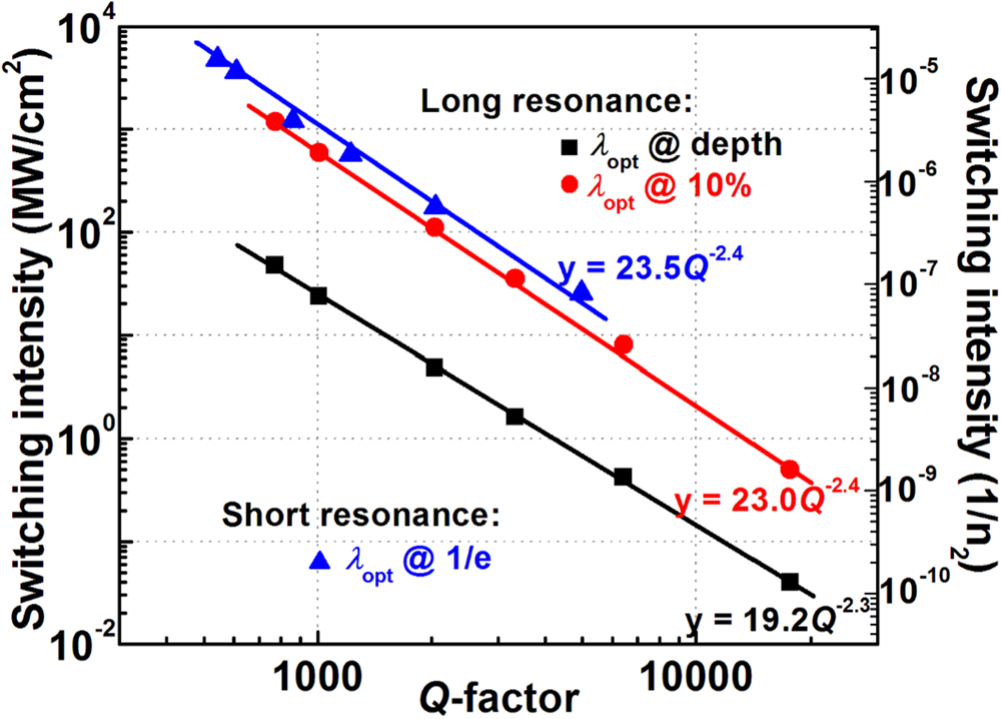
Optical incident intensity for the switching of optical switching/bistability devices based on 3-pair-grating layers for various *Q*-factors. The solid lines are fitting with their noted equations.

Figure 6 shows the calculated switching intensity (in the units of MW/cm^2^ and 1/n_2_) for various *Q*-factors. The fitting equation and the line for the switching intensity are also noted. It is clearly seen that the switching intensities decrease roughly as 1∕*Q*^2.4^ and 1/*Q*^2.3^ for bistability and switching behaviors, respectively. It is well known that the switching intensities of an established Lorentzian lineshape optical bistable device in photonic crystal slabs or slab waveguide gratings scale as 1/*Q*^2^ ^39,40^, where *Q* = *λ*_o_/Δ*λ*, *λ*_o_ and Δ*λ* are the resonant wavelength and full-width at half-maximum, respectively. This implies that the switching intensity based on Fano resonances decrease faster than that of the Lorentzian lineshapes. If the nonlinear characteristics of the Fano resonances are similar to that of a Lorentzian lineshape, the normalized switching intensity should be proportional to the 1/(Δ*λ*)^2^. This implies that the switching/bistability behavior of the Fano resonances is different from that of a Lorentzian lineshape and the switching intensity of the Fano resonances cannot be simply estimated from its 1/(Δ*λ*)^2^. Since the optical field is distributed over grating in the Fano resonant structure in contrast to the single grating-based Lorentzian lineshape. It may be advantageous in terms of prevention of material breakdown or unwanted nonlinear effects.

As results shown above, the significant advantage of the multilayer grating structure is that the *Q*- and *q*-factors can be tuned by tailoring the number of layers and the grating widths. Fig. S3 (see Supporting Information) shows the optical incident intensity for the switching of optical switching/bistability for various numbers of layers *N* for long (TE_0_) and short (TE_1_) resonances. These results can be estimated by combining the dependences of *Q*-factor on the number of layers *N* and of switching intensity on the *Q*-factor as shown in Figs 3 and 6, respectively. Note that only grating width *w* of 70 nm and the operating wavelengths at depth and 1/e of transmissions corresponding to the positive and negative of *q*-factors, respectively are shown here. The switching intensity decreases as number of layers *N* increases. Shown in Fig. S3(a), the switching intensity decreased almost by an order of magnitude for increased number of layers *N* from 3 to 5 for the long resonance. Whereas, the switching intensity is reduced 11 times when 5-pair device is compared to 3-pair grating structure in the same working condition for short resonance as shown in Fig. S3(b).

## Conclusion

In conclusion, the Fano resonance manipulation in the multilayer dielectric gratings has been discussed highlighting the quality and asymmetric factors of the optical spectrum. The investigated structure shows a possibility for the switching/bistability behaviors in which the switching intensity reduction dependency on a quality factor in a quantitative way can be obtained. We believe that our design and numerical investigation have been a useful guideline for the implementation of Fano resonant configurations for applications in optical devices, especially in switching/bistability.

## Electronic supplementary material

Supporting information
