# Supplementary material for: Controlling Fano resonances in multilayer dielectric gratings towards optical bistable devices
Source: Sci Rep. 2018 Nov 6;8:16404. doi: 10.1038/s41598-018-34787-9 (PMC6219597; doi:10.1038/s41598-018-34787-9)
Supplement: Supplementary file 1 — Supporting information [file 41598_2018_34787_MOESM1_ESM.doc]

**Supporting information**

**Controlling Fano resonances in multilayer dielectric gratings towards optical bistable devices**

Thu Trang Hoang^1,2^, Quang Minh Ngo^1,2*^, Dinh Lam Vu^1,2^, and Hieu P. T. Nguyen^3^

^1^ Institute of Materials Science, Vietnam Academy of Science and Technology, 18 Hoang Quoc Viet, Cau Giay, Hanoi, Vietnam

^2^ Graduate University of Science and Technology, Vietnam Academy of Science and Technology, 18 Hoang Quoc Viet, Cau Giay, Hanoi, Vietnam

^3^ Department of Electrical and Computer Engineering, New Jersey Institute of Technology, Newark, New Jersey 07102, USA

^*^Corresponding author: minhnq@ims.vast.ac.vn

**Additional data:**

**
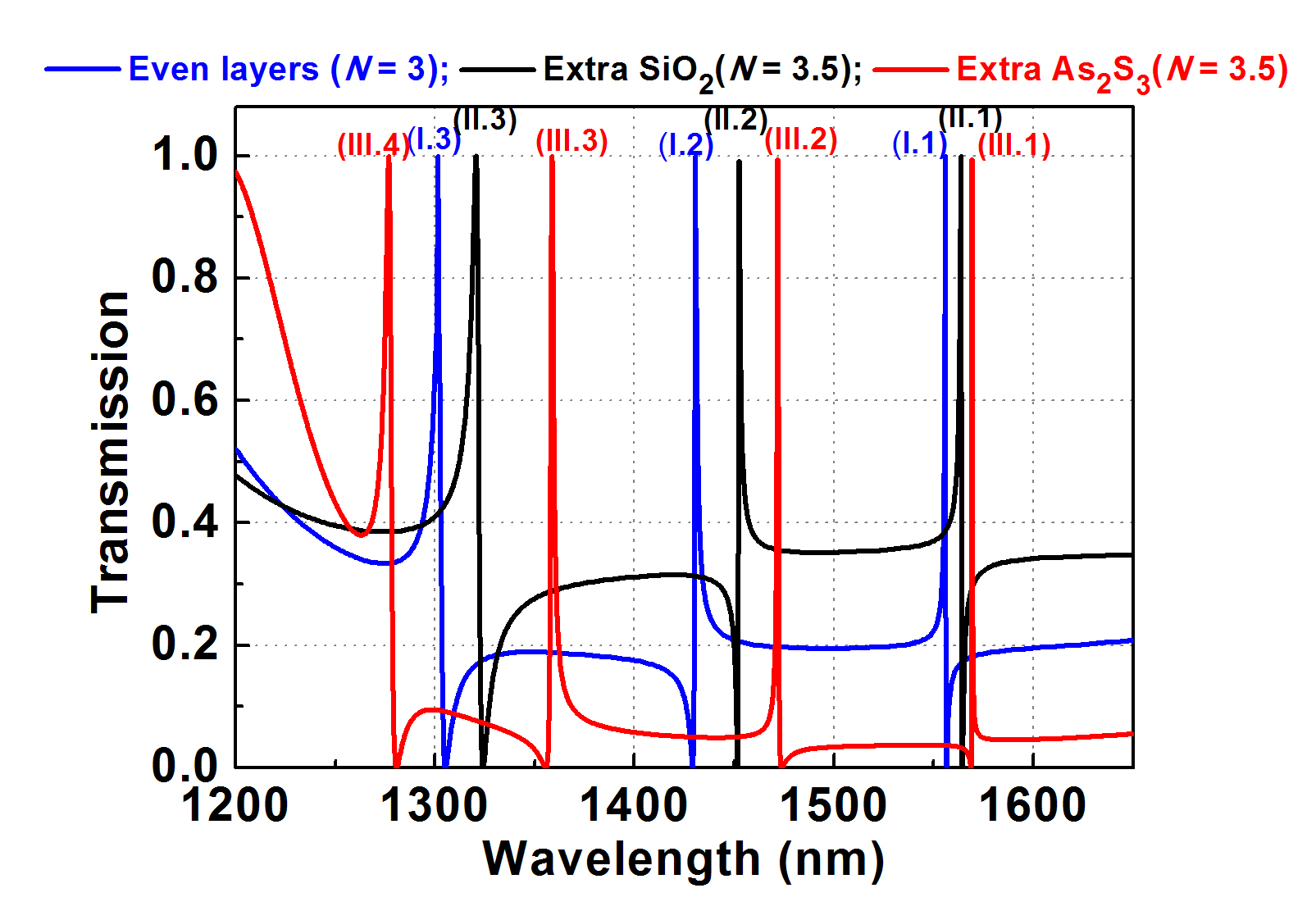
**

**Figure S1.** The full resonant spectra of the structure as depicted in Fig. 2 for even layers (*N* = 3, blue curve), odd layers (*N* = 3.5, extra SiO_2_, black curve), and odd layers (*N* = 3.5, extra As_2_S_3_, red curve). The grating width *w* = 70 nm is used for these calculations.

**Table S1.** The field profiles at resonant peaks shown in Fig. S1.

| **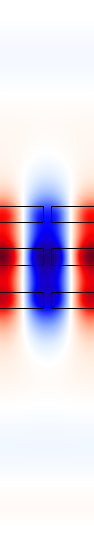** | **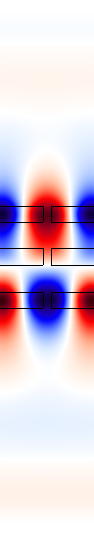** | **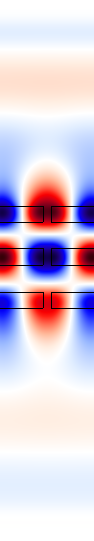** |
| --- | --- | --- |
| (I.1) λ_o_ = 1556.0 nm; *Q*-factor = 3926; TE_0_-like mode | (I.2) λ_o_ = 1430.4 nm; *Q*-factor = 1222; TE_1_-like mode | (I.3) λ_o_ = 1302.8 nm; *Q*-factor = 370.8; TE_2_-like mode |
| 1. Even layers (*N* = 3) | | |

| **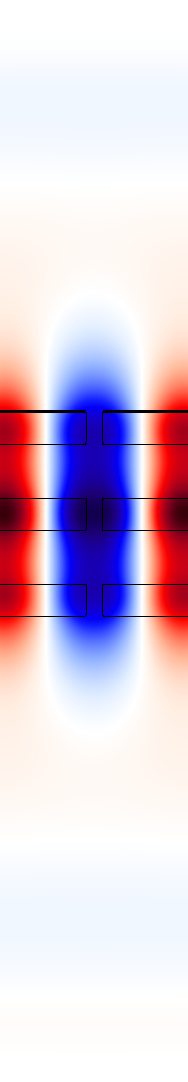** | **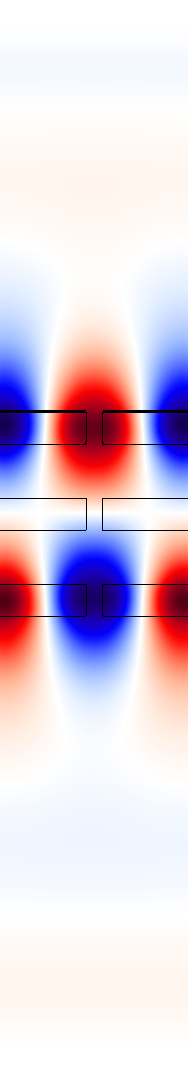** | **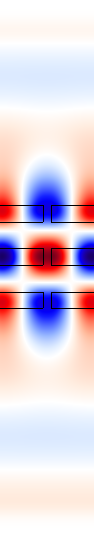** |
| --- | --- | --- |
| (II.1) λ_o_ = 1563.9 nm; *Q*-factor = 4446; TE_0_-like mode | (II.2) λ_o_ = 1452.3 nm; *Q*-factor = 1560; TE_1_-like mode | (II.3) λ_o_ = 1321.8 nm; *Q*-factor = 404; TE_2_-like mode |
| (b) Odd layers (*N* = 3.5) Extra SiO_2_ | | |

| **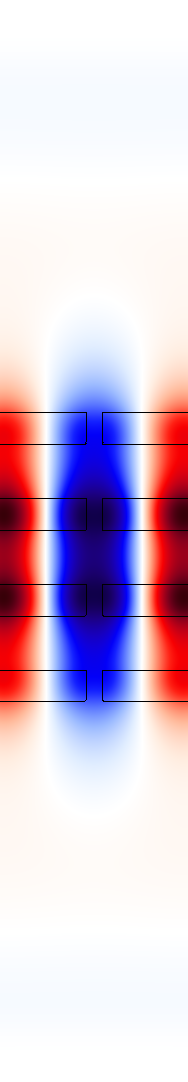** | **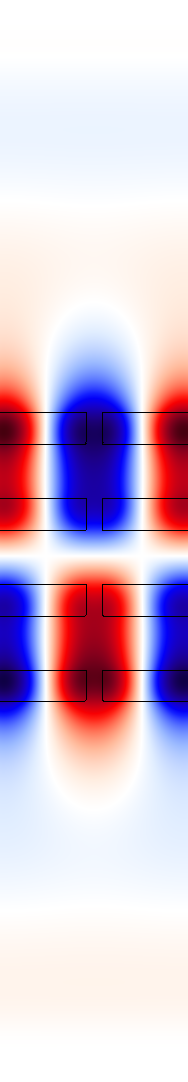** | **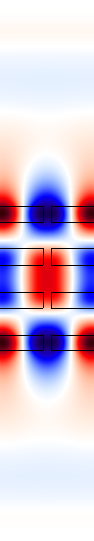** | **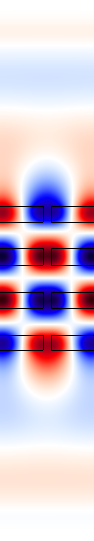** |
| --- | --- | --- | --- |
| (III.1) λ_o_ = 1569.3 nm; *Q*-factor = 7680; TE_0_-like mode | (III.2) λ_o_ = 1472.0 nm; *Q*-factor = 1915; TE_1_-like mode | (III.3) λ_o_ =1358.5 nm; *Q*-factor = 874; TE_2_-like mode | (III.4) λ_o_ = 1277.7 nm; *Q*-factor = 421; TE_3_-like mode |
| 1. Odd layers (*N* = 3.5) Extra As_2_S_3_ | | | |

| **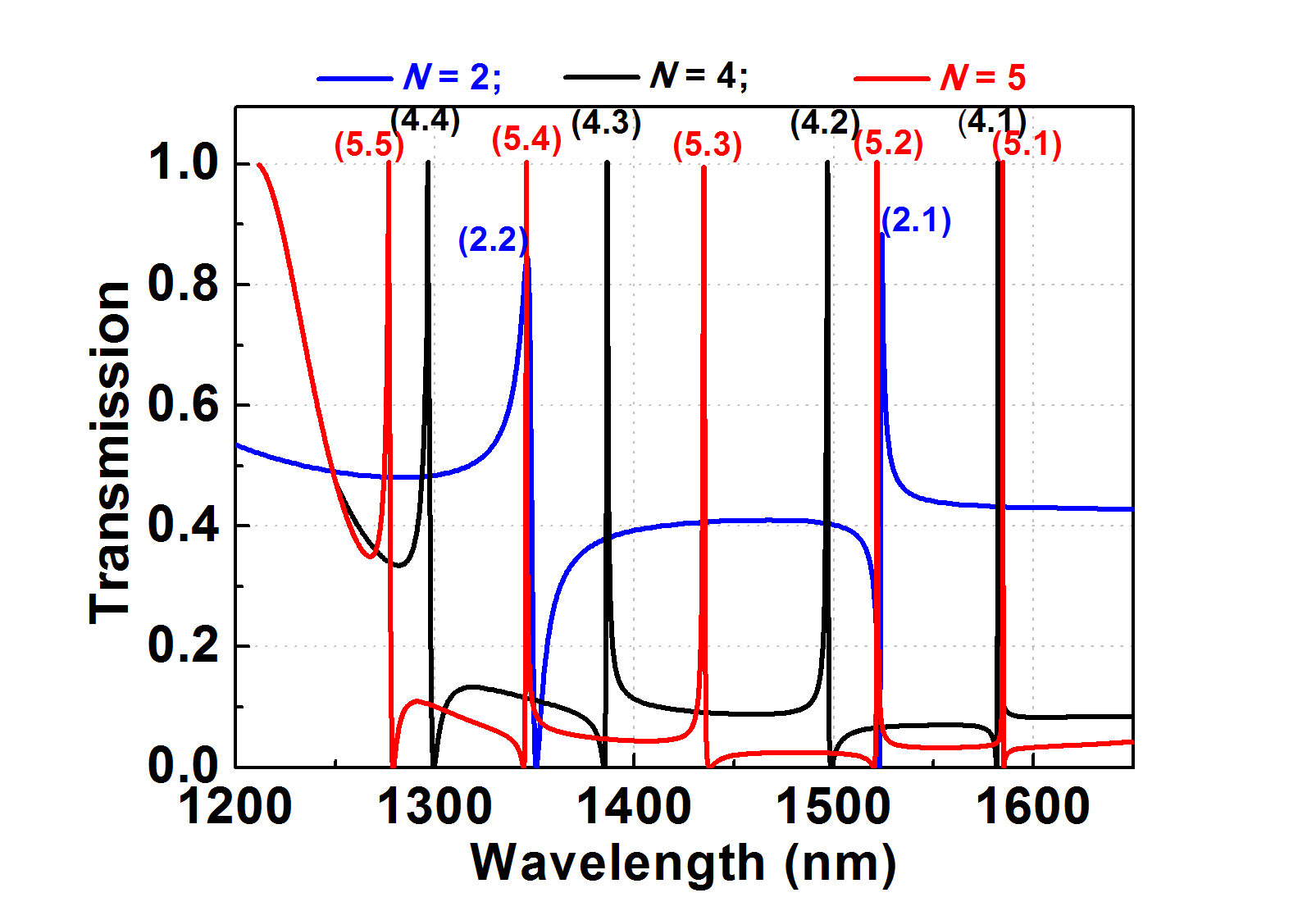** |
| --- |
| **Figure S2.** Similar to Fig. S1 but with different stack numbers *N* = 2 (blue curve), *N* = 4 (black curve), and *N* = 5 (red curve). |

**Table S2. The field profiles at resonant peaks shown in Fig. S2.**

| **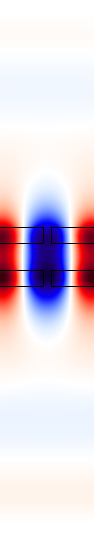** | **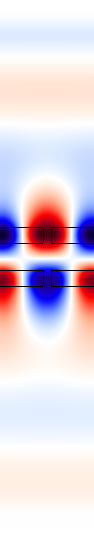** |
| --- | --- |
| (2.1) λ_o_ = 1523.5 nm; *Q*-factor = 1487.2; TE_0_-like mode | (2.2) λ_o_ = 1348.6 nm; *Q*-factor = 297.3; TE_1_-like mode |
| (a) *N* = 2 | |

| **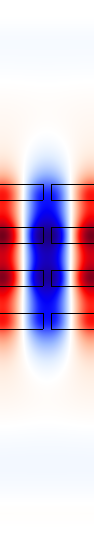** | **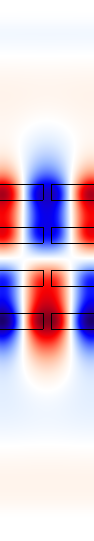** | **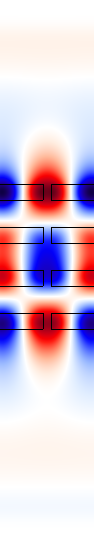** | **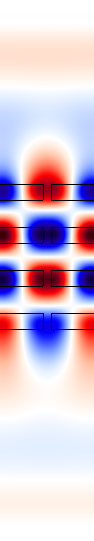** |
| --- | --- | --- | --- |
| (4.1) λ_o_ = 1582.0 nm; *Q*-factor = 5445.3; TE_0_-like mode | (4.2) λ_o_ = 1497.5 nm; *Q*-factor = 1643.8; TE_1_-like mode | (4.3) λ_o_ = 1386.1 nm; *Q*-factor = 1029.7; TE_2_-like mode | (4.4) λ_o_ = 1297.1 nm; *Q*-factor = 483.1; TE_3_-like mode |
| (b) *N* =4 | | | |

| **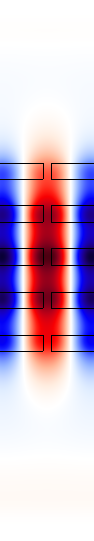** | **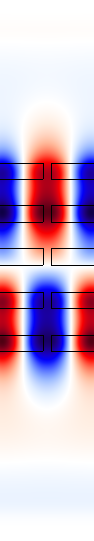** | **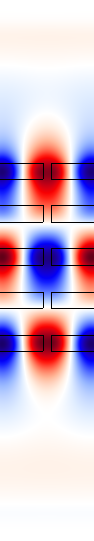** | **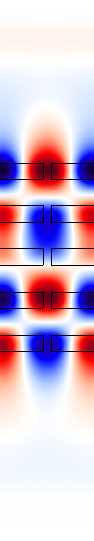** | **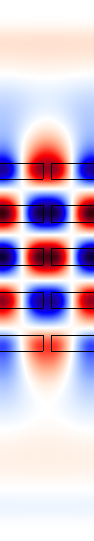** |
| --- | --- | --- | --- | --- |
| (5.1) λ_o_ = 1586.7 nm; *Q*-factor = 10684.3; TE_0_-like mode | (5.2) λ_o_ = 1521.5 nm; *Q*-factor = 2711.3; TE_1_-like mode | (5.3) λ_o_ = 1434.9 nm; *Q*-factor = 1457.1; TE_2_-like mode | (5.4) λ_o_ = 1345.7 nm; *Q*-factor = 1095.4; TE_3_-like mode | (5.5) λ_o_ = 1277.3 nm; *Q*-factor = 603.9; TE_4_-like mode |
| (c) *N* = 5 | | | | |

| 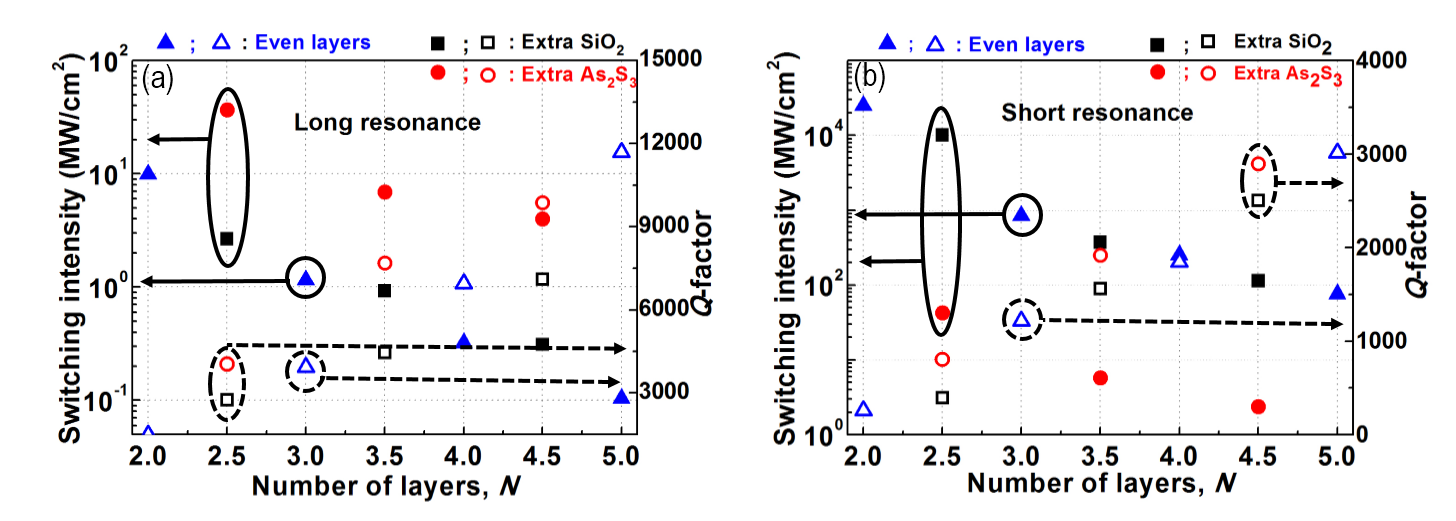 |
| --- |
| **Figure S3**. Estimated optical incident intensity for the switching of optical switching/bistability for various number of layers *N* and grating width *w* of 70 nm for the (a) long (TE_0_) and (b) short (TE_1_) resonances. The operating wavelengths at depth and 1/e of transmissions corresponding to the positive and negative of *q*-factors, respectively. |
